# Prefrontal meta-control incorporating mental simulation enhances the adaptivity of reinforcement learning agents in dynamic environments

**DOI:** 10.3389/fncom.2025.1559915

**Published:** 2025-03-27

**Authors:** JiHun Kim, Jee Hang Lee

**Affiliations:** ^1^Graduate School of AI and Informatics, Sangmyung University, Seoul, Republic of Korea; ^2^Department of Human-Centered AI, Sangmyung University, Seoul, Republic of Korea; ^3^Center for Neuroscience-Inspired AI, Institute for Advanced Intelligence Study, Daejeon, Republic of Korea

**Keywords:** prefrontal meta-control, mental simulation, model-free learning strategy, model-based learning strategy, neuroscience of reinforcement learning, reinforcement learning agents

## Abstract

**Introduction:**

Recent advances in computational neuroscience highlight the significance of prefrontal cortical meta-control mechanisms in facilitating flexible and adaptive human behavior. In addition, hippocampal function, particularly mental simulation capacity, proves essential in this adaptive process. Rooted from these neuroscientific insights, we present *Meta-Dyna*, a novel neuroscience-inspired reinforcement learning architecture that demonstrates rapid adaptation to environmental dynamics whilst managing variable goal states and state-transition uncertainties.

**Methods:**

This architectural framework implements prefrontal meta-control mechanisms integrated with hippocampal replay function, which in turn optimized task performance with limited experiences. We evaluated this approach through comprehensive experimental simulations across three distinct paradigms: the two-stage Markov decision task, which frequently serves in human learning and decision-making research; *stochastic GridWorldLoCA*, an established benchmark suite for model-based reinforcement learning; and a *stochastic Atari Pong* variant incorporating multiple goals under uncertainty.

**Results:**

Experimental results demonstrate *Meta-Dyna*'s superior performance compared with baseline reinforcement learning algorithms across multiple metrics: average reward, choice optimality, and a number of trials for success.

**Discussions:**

These findings advance our understanding of computational reinforcement learning whilst contributing to the development of brain-inspired learning agents capable of flexible, goal-directed behavior within dynamic environments.

## 1 Introduction

The integration of reinforcement learning (RL) with deep learning architectures, called Deep RL, has accomplished unprecedented performance across numerous domains. Whilst the majority of RL implementations have relied upon model-free (MF) principles, there exists an expanding collection of model-based (MB) algorithms that aim to leverage enhanced sample efficiency and adaptive capacity. However, recent benchmark analyses by Wan et al. (2022) challenge the assumption of MB supremacy over MF approaches. Recent findings in decision neuroscience suggest compelling evidence that the fundamental principle underlying human RL resides in meta-control mechanisms—specifically, the arbitration between MB and MF learning strategies, which dynamically adjusts based on environmental complexity and cost-benefit trade-offs (Kool et al., 2017; Kim et al., 2023), highlighting its crucial role in learning efficiency (Daw and Dayan, 2014).

Neuroscientific research in RL indicates a dual-process system consisting of MF and MB learning strategies: MF learning facilitates habitual behavior acquisition through reward prediction error (RPE), whilst MB learning enables goal-directed behavior through state prediction error (SPE), as established by Daw et al. (2005). Although state-of-the-art RL implementations predominantly adopt MF principles, MB approaches gain increasing attention due to their enhanced sample efficiency and adaptive capacity. Moreover, Dayan and Berridge (2014) demonstrated that MB learning augments MF approaches through cognitive prediction via environmental representation, thereby optimizing reward maximization with respect to computational efficiency and cognitive resource allocation (Dayan and Berridge, 2014).

However, despite scant research focus on MB approaches, their purported advantages in sample efficiency and adaptive capacity relative to MF implementations remain contentious (Wan et al., 2022). This observation precipitates critiques that MB RL does not consistently outperform MF RL, particularly in tasks that humans successfully achieve with relative ease. When environmental models fail to acquire complete state transition probabilities, performance becomes sub-optimal due to prolonged training requirements, as demonstrated by Bansal et al. (2017). Additionally, in experimental paradigms prevalent in neuroscience and cognitive psychology, such as the two-stage Markov decision task (MDT), purely MB strategies exhibit partial alignment with human behavior but fail to achieve complete correspondence, as established by Daw et al. (2011). These findings suggest inherent limitations in purely MB learning strategies, even within simple task environments that humans readily master.

In effect, research indicates that the fundamental principle underlying RL in the human brain centers on meta-control—specifically, the arbitration between MB and MF RL strategies (Lee et al., 2014; Daw et al., 2005; Abbott and Dayan, 2001). Neural correlates of State Prediction Error and Reward Prediction Error manifest in the dorsal prefrontal cortex (dlPFC) and ventral striatum, respectively. The inferior lateral prefrontal cortex (ilPFC) evaluates the relative reliability of competing learning strategies, whilst the ventromedial prefrontal cortex (vmPFC) functions as an arbitrator, implementing parallel control of MB and MF valuations (Lee et al., 2014; Dolan and Dayan, 2013). This neural architecture enables robust adaptation to environmental dynamics whilst optimizing the trade-off between performance, efficiency, and processing speed (Lee et al., 2019, 2022).

Complementary to meta-control mechanisms, mental simulation processes play a pivotal role in facilitating rapid and adaptive behavior during MB learning. Mental simulation constitutes a core mechanism through which the brain evaluates potential actions using internal environmental representations (Tolman, 1948; Daw et al., 2005). This cognitive capacity manifests even in rodent behavior, enabling novel route discovery and flexible planning under changing goal conditions (Dickinson, 1985; Tolman, 1948). Whilst step-wise mental simulation incurs substantial computational demands, it provides efficient adaptation to environmental dynamics through comprehensive state-space evaluation for optimal policy execution (Daw and Dayan, 2014).

The functional relationship between mental simulation and hippocampal replay elucidates neural mechanisms for managing these computational complexity. Hippocampal replay serves multiple functions: (i) facilitating sequential activation of hippocampal cells during rest periods (Karlsson and Frank, 2009), (ii) encoding topological structures of novel environments (Wu and Foster, 2014), and (iii) enabling goal-directed path simulation (Pfeiffer and Foster, 2013). These processes operate synergistically within the successor representation (SR) framework.

The SR framework extends predictive abilities through replay mechanisms, facilitating offline training via simulated experiences (Russek et al., 2017; Momennejad et al., 2017). This integration enables rapid action evaluation whilst maintaining behavioral flexibility through offline learning processes (Sutton, 1990; Mattar and Daw, 2018). Past research demonstrates that replay transcends mere experiential recapitulation, enabling novel trajectory construction (Gupta et al., 2010). Moreover, human studies indicate that replay events manifest abstract structural knowledge of acquired tasks (Liu et al., 2019). Notably, replay disruption impairs learning in contexts requiring history-dependent inference (Jadhav et al., 2012).

Based upon these neuroscientific insights, we present *Meta-Dyna*, RL architecture. Extending the *Dyna-Q* framework (Sutton and Barto, 2018), *Meta-Dyna* implements prefrontal meta-control mechanisms, providing an algorithmic model of arbitration between MF and MB learning strategies (Lee et al., 2014). We in addition enhance the MB component through integration of deep learning-based environmental modeling, enabling replay capacity via roll-out methodology. This architecture synthesizes meta-control mechanisms with mental simulation capabilities, thereby aiming to enhance RL agents' performance, environmental adaptation, and behavioral flexibility.

## 2 Preliminaries

### 2.1 Mental simulation and *Dyna* architecture

RL constitutes a framework where an agent acquires optimal action selection through environmental interaction to maximize future rewards. The environment, which is modeled as a Markov Decision Process (MDP), comprises a tuple < **S**, **A**, *R, T*, γ, **S'** >. This tuple includes a set of states *S*, actions *A*, rewards *R*, state-action-state transition probability *T*, discount factor γ and transitions to the next states **S'**. Within an MDP, the probability of transition to the subsequent state relies solely upon the current state and action, irrespective of antecedent history (by Markov property). The agent endeavors to ascertain a policy π that stipulates actions for each state to maximize cumulative rewards.

*Dyna-Q* (Sutton, 1990), which amalgamates direct experiential learning with simulated experience planning, employs a planning component that enables the agent to augment its knowledge via a learnt environmental model rather than exclusively through actual experiences. The *Dyna-Q* planning process involves training an environmental model that predicts subsequent states and rewards, based on current states and actions. These simulated data facilitate Q-learning implementation, expediting optimal policy convergence. The acceleration of convergence correlates positively with increased planning steps, thus demonstrating the efficacy of incorporating simulation-based planning mechanisms into RL (Sutton, 1990; Sutton and Barto, 2018).

The brain exhibits analogous mechanisms to *Dyna*, utilizing both direct experience and simulated trajectories in choice evaluation (Momennejad et al., 2017). Through this simulation capacity, humans and animals successfully identify and circumvent suboptimal outcome pathways (Allen et al., 2020; Miller et al., 2017).

In this sense, mental simulation in the brain can be computationally implemented through various approaches, including the *Dyna* model architecture. Sutton (1990) introduced *Dyna* as an integrated architecture for learning, planning, and reacting, which simulates experience offline to update predictions. This approach mirrors how the brain might use mental simulation and replay to enhance learning. Nonetheless, whilst *Dyna* reflects how the brain improves learning via replay, recent studies have revealed its limitations, including decreased learning efficiency in real-world environments (Barkley and Fridovich-Keil, 2024). To that end, researchers have developed several improvements. These include data-driven inventory management using *Dyna-Q* (Qu et al., 2025) and out-of-distribution (OOD) data filtering, which enhances model reliability (Li et al., 2024). These optimisation efforts have expanded into various fields, such as industrial automation (Dong et al., 2020; Liu and Wang, 2021; Budiyanto and Matsunaga, 2023; Samaylal, 2024) and energy management (Saeed et al., 2024; Ghode and Digalwar, 2024; Liu et al., 2024, 2025).

The relationship between mental simulation and *Dyna* manifests particularly within the successor representation (SR) framework. The SR, which accommodates various hybrid implementations, includes “SR-*Dyna*” that employs either simulation or replay for offline SR updating (Russek et al., 2017; Momennejad et al., 2017). This offline updating process within *Dyna* mirrors the brain's adoption of hippocampal replay for goal-directed path simulation and construction.

A fundamental characteristic that bridges mental simulation and *Dyna* lies in their offline planning functionality. The brain's utilization of mental simulation during rest periods for enhanced learning and decision-making parallels *Dyna*'s deployment of simulated experience for offline value estimation updates. This correspondence suggests that *Dyna* captures essential aspects of the brain's flexible planning implementation through mental simulation (Mattar and Daw, 2018).

### 2.2 *Dyna* architecture and prefrontal meta-control in the human brain

Nevertheless, a fundamental question persists with regards to the harmonization of the dual systems within *Dyna*—namely, MB and MF components—toward bringing about optimal policy. Recent advances in decision neuroscience, which illuminate the arbitration control mechanisms that govern multiple learning strategies, inspire us. These advances proffer a resolution to this conundrum. These mechanisms, which specifically contain MB and MF RL paradigms, demonstrate remarkable efficacy in strategy reconciliation (Lee et al., 2014; Daw et al., 2005; Abbott and Dayan, 2001).

The arbitration control mechanism proves integral to decision-making optimisation, particularly in contexts where the relative appropriateness of MB vs. MF strategies exhibits variability. Lee et al. (2014), which presents neural evidence for an arbitration mechanism, demonstrates that the degree of control exerted by these dual strategies depends upon their respective prediction error (PE) reliability.

The Reward Prediction Error (RPE), which serves to compute the reliability of the MF strategy (*Rel*_*MF*_), is calculated through Temporal Difference error (TD-Error). The mathematical formulation for this error is expressed as $RPE=r_{t}+\gamma Q_{MF}\left(s^{\prime },a^{\prime };\theta_{MF}\right)-Q_{MF}\left(s,a;\theta_{MF}\right)$. Conversely, the State Prediction Error (SPE), which determines the reliability of the MB strategy (*Rel*_*MB*_), is formulated as *SPE* = 1−*T*(*s, a, s*′), where *T* denotes the state-action-state transition probabilities defined in Equation 1.


(1) $$T(s,a,s^{\prime })=\{\begin{array}{ll} P_{s}+\gamma (1-P_{s}) & if\;P_{s}==P_{s^{\prime }}\\ P_{s}\times (1-\gamma ) & \text{otherwise}. \end{array}$$

The probability of MB control (*P*_*MB*_) is derived from *Rel*_*MB*_ and *Rel*_*MF*_, which are computed through Bayesian and non-Bayesian approaches, respectively (Li et al., 2011; Le Pelley, 2004; Sutton, 1992; Krugel et al., 2009; Pearce and Hall, 1980). The pseudo-level formulations are presented in Equation 2.


(2) $$\begin{array}{l} \begin{array}{c} Rel_{MF}=Pearce\;Hall\left(RPE|PE\right),\\ Rel_{MB}=Bayesian\left(SPE|PE\right). \end{array} \end{array}$$

For the MB reliability (*Rel*_*MB*_), the Dirichlet distribution parameters—mean *E*(*Dir*_*i*_) and variance *V*(*Dir*_*i*_)—are employed, where the subscript *i* indicates three distinct State Prediction Error (SPE) categories: negative, positive, and zero prediction errors. These categorical boundaries are established through a tolerance threshold ω, such that *PE* < ω constitutes negative error, *PE* > ω represents positive error, and values within this interval denote zero error.

The probability of utilizing MB learning strategies (*P*_*MB*_) is determined via the arbitration mechanism, which adopts reliability (Equation 3).


(3) $$\begin{array}{l} \;\alpha =\frac{A_{\alpha }}{\left(1+exp(B_{\alpha }*Rel_{MF})\right)},\\ \;\beta =\frac{A_{\beta }}{\left(1+exp(B_{\beta }*Rel_{MB}\right)},\\ P_{MB}=P_{MB}+\alpha *(1-P_{MB})-\beta *P_{MB}. \end{array}$$

Through this computational framework, MB and MF values facilitate meta-control implementation. Finally they are concurrently applied in action selection through the weighting factor *P*_*MB*_.

The theoretical framework described above exhibits strong correspondence with the neural substrates that underlie these computational processes. The bilateral inferior lateral prefrontal cortex regions, which encode MB and MF signal reliability, function in concert with the anterior cingulate cortex, which integrates reliability differentials to mediate arbitration (Lee et al., 2014). This reliability-driven arbitration mechanism determines strategic dominance, thereby facilitating dynamic environmental adaptation. Furthermore, the arbitration process receives support from additional neural structures: the ventral and dorsal striatum, which encode Reward Prediction Error (RPE), and the temporo-parietal cortex, which encodes State Prediction Error (SPE).

This neurobiological evidence substantiates that the fundamental question regarding dual-process amalgamation can be resolved, which has precipitated the development of neuroscience-inspired algorithmic frameworks that extend beyond the classical *Dyna* architecture.

## 3 *Meta-Dyna*: a neuroscience-inspired RL architecture

**Figure 1:**
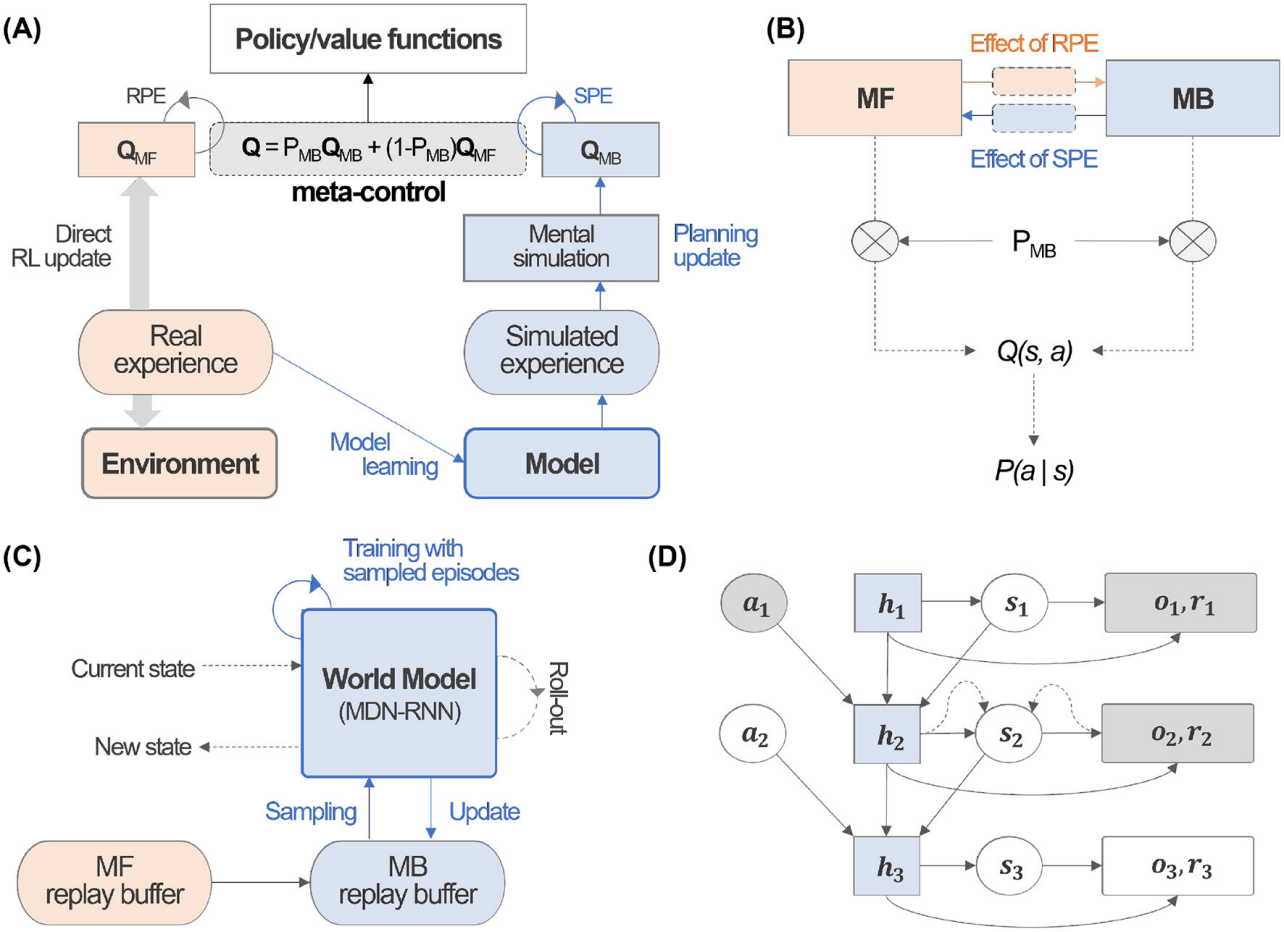
*Meta-Dyna* overview. **(A)** Schematic architecture of *Meta-Dyna*. **(B)** An illustration of prefrontal meta-control, which arbitrates between MB and MF learning strategies. **(C)** A schematic representation of the world model that enables mental simulation. **(D)** A demonstration of the roll-out process, which implements mental simulation for given state-action pairs.

Within the context of recent RL, we propose *Meta-Dyna*, which constitutes a neuroscience-inspired algorithmic framework that extends the foundational *Dyna-Q* architecture (Figure 1). This novel approach implements dual learning processes – MB planning and MF Q-learning—whilst incorporating an arbitration mechanism for implementing the prefrontal meta-control. Moreover, owing to the inherent properties of the *Dyna* architecture, it facilitates mental simulation through the MB learning system. The framework, which embodies current neuroscientific understanding of cognitive processes, demonstrates how the brain synthesizes diverse learning strategies through mental simulation and experiential replay.

### 3.1 Overview

As described, *Dyna-Q* integrates Q-learning with planning mechanisms, which update Q-values through MB processes. The architecture employs planning through an environmental model that agents acquire via direct experiential interaction. *Meta-Dyna*, which extends this foundational architecture, implements dual Q-value systems: MB planning (*Q*_*MB*_) and MF Q-learning (*Q*_*MF*_).

*Meta-Dyna* distributes received state information to both real and model experience buffers. During the *Q*_*MF*_ update process, the world model incorporates training using a superset, which comprises both simulated and real experiences. The trained model subsequently generates simulated experiences through a recursive process. These experiences facilitate mental simulation, which ultimately updates *Q*_*MB*_.

The prefrontal meta-control arbitrator computes the MB probability (*P*_*MB*_) using reliability, which derive from Prediction Errors of each learning system. This meta-control component computes an integrated Q-value through weighted summation: *P*_*MB*_ for *Q*_*MB*_ and 1−*P*_*MB*_ for *Q*_*MF*_ (Figure 1A). The complete processes are detailed in Algorithm 1.

**Algorithm 1:** *Meta-Dyna* learning process.

1: Initialize *Q*_*MF*_, *Q*_*MB*_, World Model *M*, and replay buffer *RB*_*MF*_, *RB*_*MB*_
2: for each episode **do**
3: for each time step **do**
4: Select action *a*_*t*_ based on integrated *Q* values
5: Execute *a*_*t*_ and observe *s*_*t*+1_ and *r*_*t*+1_
6: Store (*s*_*t*_, *a*_*t*_, *r*_*t*+1_, *s*_*t*+1_) in *RB*_*MF*_
7: end **for**
8: Train *Q*_*MF*_ using experiences from *RB*_*MF*_
9: Train *M* using experiences from *RB*_*MB*_
10: Compute RPE and SPE
11: for *i* = 1 to *n* **do**
12: Generate simulated experience using *M*
13: Train *Q*_*MB*_ using simulated experience
14: end **for**
15: Update reliability and calculate *P*_*MB*_
16: Integrate *Q*_*MF*_ and *Q*_*MB*_ using *P*_*MB*_
17: end **for**

### 3.2 Dual Q-value system for MB and MF

*Meta-Dyna* incorporates a dual Q-value system that implements MB and MF learning strategies. The *Meta-Dyna* architecture comprises one main inference network and three component networks: two Q-networks for MB and MF learning strategies, and a world model for simulating environmental dynamics (Figure 1A).

The main inference network combines outputs from the component networks, which guide decision-making processes. This main network operates under the governance of the prefrontal meta-control framework. The two Q-networks independently learn distinct behavioral patterns: habitual behaviors (MF) and goal-directed behaviors (MB). The MF Q-network learns habitual behaviors through reward signals from real experience, which utilizes standard Q-learning methods. Meanwhile, the MB Q-network learns from the world model to perform goal-directed planning. The world model learns to predict the environment's structure, which includes future states and rewards, thereby enabling the generation of simulated data for planning.

Through the separation of Q-values into MF (*Q*_*MF*_) and MB (*Q*_*MB*_) components, *Meta-Dyna* enables independent acquisition of habitual and goal-directed strategies. This architectural distinction implements a framework, which enables the agent to adaptively favor between MB and MF strategies based on their respective reliability, particularly in response to environmental dynamics (Figure 1B).

We note that two distinct implementations of *Meta-Dyna* were presented: a tabular version and a neural network (NN) variant. This architectural flexibility derives from the framework's generalisable principles, which enable deployment across diverse environmental contexts. We here employ MDN-RNN as the NN variant, specifically for its ability to handle uncertainty and model probabilistic environmental dynamics, aligning with *Meta-Dyna's* goal of generalization (Ha and Schmidhuber, 2018). MDN-RNN combines Mixture Density Networks with recurrent neural networks, enabling it to output probability distributions rather than deterministic predictions. This approach is particularly valuable in environments that are stochastic in nature, as it allows the model to capture inherent randomness and discrete random events.

As demonstrated in Ha and Schmidhuber (2018), using MDN-RNN helps prevent the agent from exploiting imperfections in the world model by introducing controlled uncertainty through temperature parameters. This makes it more difficult for agents to find adversarial policies that might work in the model but fail in actual environments. In addition, due to its generative model characteristics, it serves as an excellent candidate for mental simulation, which can foresee future states through the roll-out functionality. It showed how their world model (using MDN-RNN) can generate hypothetical scenarios and environments that the agent can interact with. They specifically created virtual environments generated by the MDN-RNN where agents could train entirely inside these simulated environments before transferring policies to actual environments. The concept of using the model to “*foresee future states*” through roll-outs is central to their approach, as they demonstrate in both the CarRacing and VizDoom experiments where the agent learns policies by simulating potential futures within the generated environment. The complete architectural framework is presented in Figure 1A.

### 3.3 Prefrontal meta-control in *Meta-Dyna*

#### 3.3.1 Prediction errors and reliability

A key ingredient of prefrontal meta-control lies in the computation of reliability for both MB and MF strategies, which derive from their respective Prediction Errors: SPE and RPE. As previously established, RPE was computed as the temporal difference (TD) error of the *Q*_*MF*_ network, aligned with its canonical formulation (Lee et al., 2014). The computation of SPE, however, exhibited distinct methodologies contingent upon the state space characteristics—discrete or continuous.

In discrete state spaces, SPE computation employed the same computational model for learning state transition probabilities as that established in Lee et al. (2014). Within continuous state spaces, which was known to be intractable for traditional state transition probability methods, we implemented the Mixture Density Network-Recurrent Neural Network (MDN-RNN). We adopted its Gaussian Mixture Model (GMM) outputs as components for computing the SPE. The GMM generated multiple Gaussian distributions, which were characterized by parameters μ_*k*_ and σ_*k*_, representing the distribution of potential subsequent states.

The standard deviation σ_*k*_ quantifies the predictive uncertainty of subsequent states. An increased magnitude of σ_*k*_ indicates elevated uncertainty, which indicates diminished predictive accuracy. For continuous state spaces, we formulate SPE using the distributional parameters μ_*k*_ and σ_*k*_ as follows:


(4) $$\begin{array}{l} SPE=\frac{\mu }{\sigma },where\;\mu ,\sigma from\text{ GMM}. \end{array}$$

These Prediction Errors facilitated the computation of reliability measures for MB and MF strategies. The arbitration mechanism utilizes these reliability values, which modulate the strategic balance dynamically, in accordance with the prefrontal meta-control framework.

#### 3.3.2 Meta-control leading to decision making

As described, *Meta-Dyna* enables independent acquisition of Q-values through MB and MF Q-networks. This separation facilitates distinct learning trajectories, which characterize habitual and goal-directed behaviors within each Q-network. The integration of MB and MF Q-networks proceeds through a weighted mechanism, which derives from their computed reliability indices.

The reliability determines the probability (*P*_*MB*_) of selecting the MB Q-network. The Q-values from *Q*_*MB*_ and *Q*_*MF*_ are integrated through weighted summation, which employs *P*_*MB*_ and (1−*P*_*MB*_) as their respective coefficients. In the tabular implementation, this integration process comprises multiplicative operations between the Q-table values of MB and MF Q-networks with their corresponding probabilities, *P*_*MB*_ and (1−*P*_*MB*_), to update the main Q-table for inference:


(5) $$\begin{array}{l} Q\left(s,a\right)=P_{MB}\times Q_{MB}\left(s,a\right)+\left(1-P_{MB}\right)\times Q_{MF}\left(s,a\right). \end{array}$$

In the neural network implementation, this integration process applies to the Q-network parameters. The main network, which incorporates the integrated Q-values, guides action selection through an ϵ-greedy policy.

Through dynamic modulation of MB and MF Q-network integration based on their respective reliability, *Meta-Dyna* implements adaptive behavior in response to environmental dynamics, which harnesses the complementary strengths of both Q-networks.

### 3.4 Mental simulation in *Meta-Dyna*

#### 3.4.1 World model learning environmental dynamics

The world model which learns environmental structure employs a Mixture Density Network-Recurrent Neural Network (MDN-RNN) architecture for sequential modeling (Ha and Schmidhuber, 2018). The MDN-RNN architecture learns sequential state-action pairs, which predict probability distributions of subsequent states and rewards. The integrated approach captures temporal dependencies through recurrent neural networks, which represent future states through probabilistic distributions via mixture density networks. Specifically, the MDN-RNN generates distributions of potential subsequent states, which are characterized by distributional parameters—means (μ) and standard deviations (σ)—that quantify expected outcomes and their associated uncertainties.

In RL, the world model serves as a simulation framework, which enables agents to evaluate hypothetical scenarios without direct environmental interaction. This methodology demonstrates particular efficacy in dynamic environments, where the MDN-RNN architecture captures latent uncertainties within the environmental dynamics. The model's predictive capabilities enhance planning processes, which facilitate efficient policy optimisation through simulated state transitions.

The world model's predictive capacity, which are integrated within the *Meta-Dyna* arbitration framework, use MDN-RNN outputs for SPE computation. The SPE, which quantifies predictive uncertainty, is mathematically defined as


(6) $$\begin{array}{l} SPE=\frac{\mu }{\sigma }, \end{array}$$

where μ and σ derive from the Gaussian distributions that characterize subsequent states.

#### 3.4.2 Architecture

In short, the MDN-RNN learns sequences of state-action pairs and predicts the probability distribution of the next state and reward. This world model, which incorporates dynamic uncertainties into decision-making processes, enhances the agent's reasoning capacity. The architecture comprises (i) recurrent neural network layers that process input sequences to capture temporal dependencies and (ii) mixture density network layers that predict distributional parameters (μ and σ) for subsequent states and rewards. The model receives PEs as inputs, which implements an error-based learning mechanism that emulates neural computation. These errors, which *Meta-Dyna* computes during the learning process, emerge naturally without external augmentation.

#### 3.4.3 Training

The training protocol involves minimizing the PEs between predicted distributions and observed subsequent states and rewards. The training data comprise state-action sequences that derive from environmental interactions. An experience replay buffer stores model-generated experiences, which enhances learning efficiency and stability.

Once trained, the world model generates simulated scenarios for training the MB policy (*Q*_*MB*_), which is analogous to Q-learning with simulated experiences. This process enables policy-model interaction through simulated state-action pair outcomes, thus facilitating efficient strategy implementation (Figures 1C, D).

### 3.5 Algorithm

The implementation of *Meta-Dyna* is contingent upon the task complexity of the environment. For tasks with manageable state-action spaces, a tabular implementation is sufficient, where *Q*_*MB*_ and *Q*_*MF*_ are represented as tables that contain values for all possible state-action pairs. The world model maintains SPE and RPE for each state-action pair.

High-dimensional tasks, which include image-based scenarios, necessitate a neural network implementation on the other hand. In this context, *Q*_*MB*_ and *Q*_*MF*_ are implemented as feed-forward networks. In high-dimensional environments such as Atari Pong, a convolutional neural network (CNN) is used as an encoder, specifically to extract spatiotemporal features from raw pixel inputs, enabling efficient mental simulation and policy adaptation under uncertainty. These extracted features serve as input for both the MB and MF components, enhancing learning efficiency in complex visual environments. The processing of high-dimensional inputs employs convolutional neural networks (CNNs) for preprocessing, which subsequently interface with feed-forward networks for Q-value representation. The world model in the neural network variant used the MDN-RNN architecture, which processes state, action, SPE, and RPE inputs to generate subsequent states and rewards.

The learning protocol included several stages shown in Algorithm 1. Initially, all components—*Q*_*MB*_, *Q*_*MF*_, and the world model—were initialized (line 1). At each timestep, the agent acquired sequences through actions derived from integrated Q-values (lines 4–7). These Markov Decision Process sequences facilitated *Q*_*MF*_ and environmental model training, whilst computing Prediction Errors (lines 8–10).

The training of *Q*_*MB*_ used simulated experiences generated by the environmental model. Planning proceeds for *n* predefined steps, where the model generated state-action pair sequences (lines 11–14). This parameter *n* balanced planning depth with model accuracy. Empirical testing established *n* = 10 as optimal, given that larger values result in diminishing returns when model accuracy converges.

The computed PEs update reliability, which subsequently modulates the integration of MB and MF learning strategies (lines 15–16). This iterative process enables *Meta-Dyna*'s adaptive learning within dynamic environments.

## 4 Experiments

**Figure 2:**
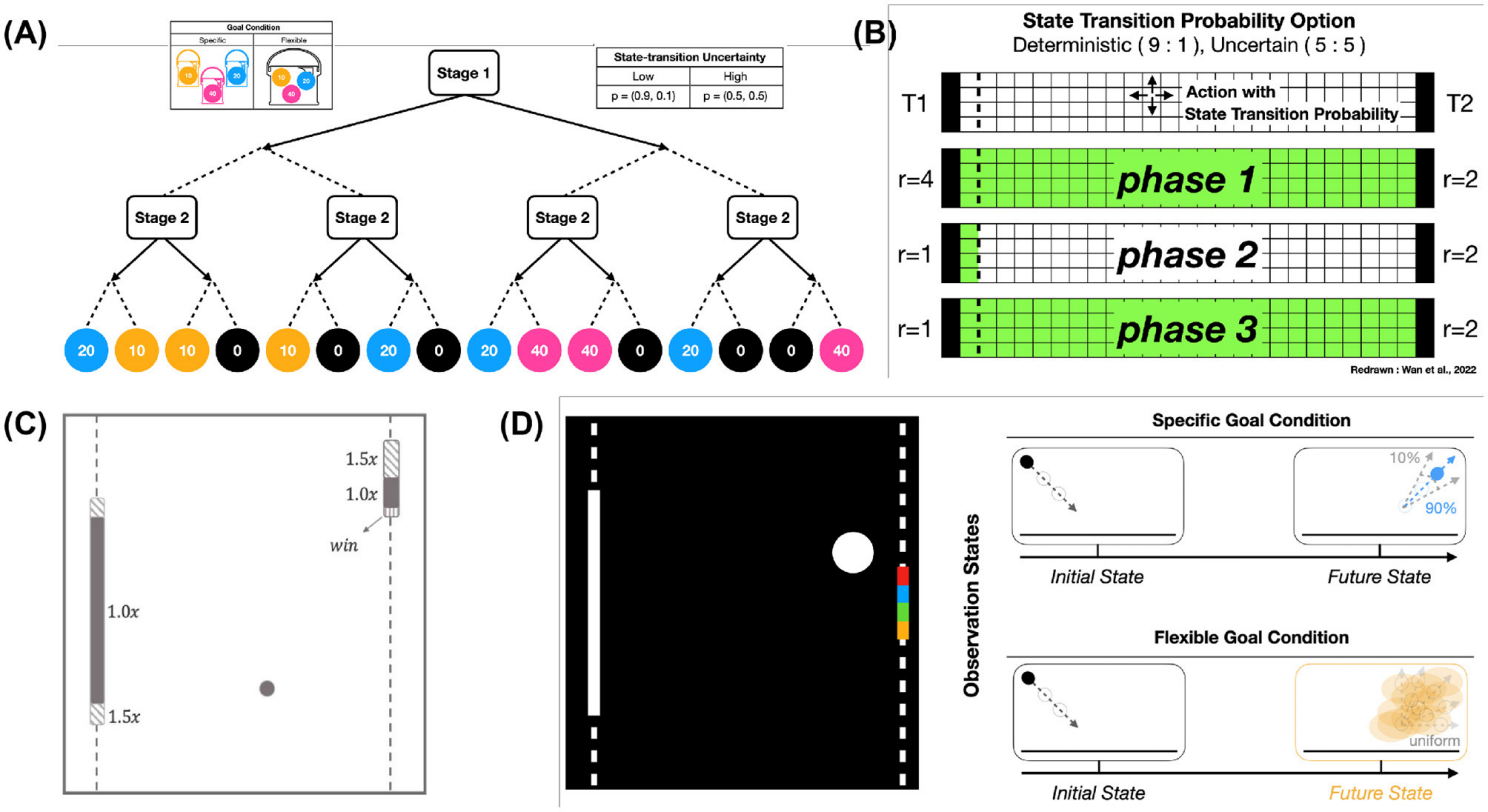
*Meta-Dyna* tasks for experiments. **(A)** Two-stage Markov decision task. **(B)** A variant of GridWorldLoca. **(C)** A dimension of Atari Pong variant (© copyright, Keramati et al., 2018). **(D)** A novel Atari Pong incorporating decision uncertainty and frequent goal changes.

To evaluate the behavioral flexibility and adaptation capacity of *Meta-Dyna*, we conducted experimental evaluations across three distinct paradigms. The first assessment employed the *Two-Stage Markov Decision Task (MDT)*, which is widely used in human decision-making research, specifically for choice behavior that is governed by MF and MB learning strategies. We subsequently developed a variant of the *GridWorldLoCA* environment to examine the rapid adaptation capacity inherent in MB strategies. Finally, to assess mental simulation coupled with prefrontal meta-control, we designed a stochastic *Atari-Pong* variant that incorporates decision-making parameters from the *Two-Stage MDT* (Figure 2).

### 4.1 Challenges: environmental uncertainty and goal condition dynamics

Our research addresses two fundamental challenges in decision-making processes that arise from recent neuroscience tasks: environmental uncertainty and goal-state dynamics. Within a MDP framework, environmental changes manifest through continuous modifications in reward conditions *R*(*s, a*). This formulation establishes that the agent's learning objective consists of achieving specific state conditions, which remains fundamental to strategy acquisition and behavioral adaptation (Lee et al., 2014, 2019; Kim et al., 2019).

Goal conditions serve as critical metrics for assessing RL agents' flexibility within dynamic environments. These conditions manifest in two forms: specific criteria that require precise reward thresholds, and flexible criteria that accommodate varied outcomes normalized to values 0, $\frac{1}{4}$, $\frac{1}{2}$, 1, which are contingent upon received rewards (Lee et al., 2014; Kim et al., 2019, 2021). This framework necessitates continuous policy re-evaluation and adaptive response refinement through iterative learning mechanisms.

An alternative representation of environmental dynamics involves modifying state transition probabilities *P*(*s*′∣*s, a*) within the MDP across specified ranges at predetermined intervals. This approach provides quantitative modeling of environmental dynamics, where state-transition probability serves as a crucial metric for uncertainty representation. The framework encompasses varying uncertainty levels: from near-deterministic outcomes with 90% probability (low uncertainty) to equiprobable outcomes of 50% (high uncertainty). These probabilistic structures reflect diverse environmental scenarios, which prove essential for addressing unpredictable changes.

This stochastic framework enables optimal action selection under dynamic conditions whilst facilitating predictive planning of future scenarios. Thus, state transition probabilities constitute fundamental factors in determining an agent's adaptive capacity within changing environments. Human decision-making demonstrates robust adaptation to varying goal conditions and state transition probabilities through prefrontal meta-control. To evaluate RL agents' adaptability, we employ MDP tasks that embody these environmental dynamics, particularly the Two-Stage MDT (Figure 2A), which incorporates both goal condition and state-transition probability variations.

These experimental paradigms comprise alternating phases of varying goal criteria and uncertainty levels, which challenge adaptive capabilities across environmental shifts. To extend these stochastic frameworks into general domains, we integrated these challenges into two distinct environments: *GridWorldLoca*, a benchmark suite for MB RL agents (Figure 2B), and a modified *Atari Pong* environment (Figure 2D). These implementations instantiate environmental uncertainty and goal-state dynamics within standardized testing paradigms.

### 4.2 Two-stage Markov decision task

#### 4.2.1 Overview

The Two-Stage MDT (Lee et al., 2014) represents a widely accepted paradigm in human decision-making research, where participants execute sequential actions to obtain color-coded tokens that correspond to specific rewards. This experimental environment consists of two distinct conditions: *Specific*– and *Flexible* Goal conditions. The former necessitates MB strategies for reward maximization, whilst the latter facilitates MF strategy utilization. We adopted this paradigm for computational evaluation of agent adaptability. Our implementation maintains fidelity with the original experimental design, incorporating both *Goal Conditions* and *State-Transition Uncertainty* parameters.

As illustrated in Figure 2A, the Goal Condition bifurcates into *Specific Goal* and *Flexible Goal* variants. Under the Specific Goal condition, the agent receives a binary reward (1.0 for required token acquisition, 0.0 otherwise). Conversely, the Flexible Goal condition implements a normalized reward spectrum 0, $\frac{1}{4}$, $\frac{1}{2}$, 1 based on token acquisition, without specific token requirements. State-Transition Uncertainty emerges in two forms: *Low* and *High*. Low uncertainty conditions encompass deterministic action execution with 0.9 probability, whilst High Uncertainty conditions exhibit equiprobable action outcomes (0.5 probability for intended and opposite actions).

#### 4.2.2 Experimental setting

The Two-Stage MDT structure, as presented in Figure 2A, comprises sequential binary actions (left/right), where the initial action results in a state representation without reward, whilst the subsequent action generates stochastic rewards based on environmental parameters before episode termination.

**Figure 3:**
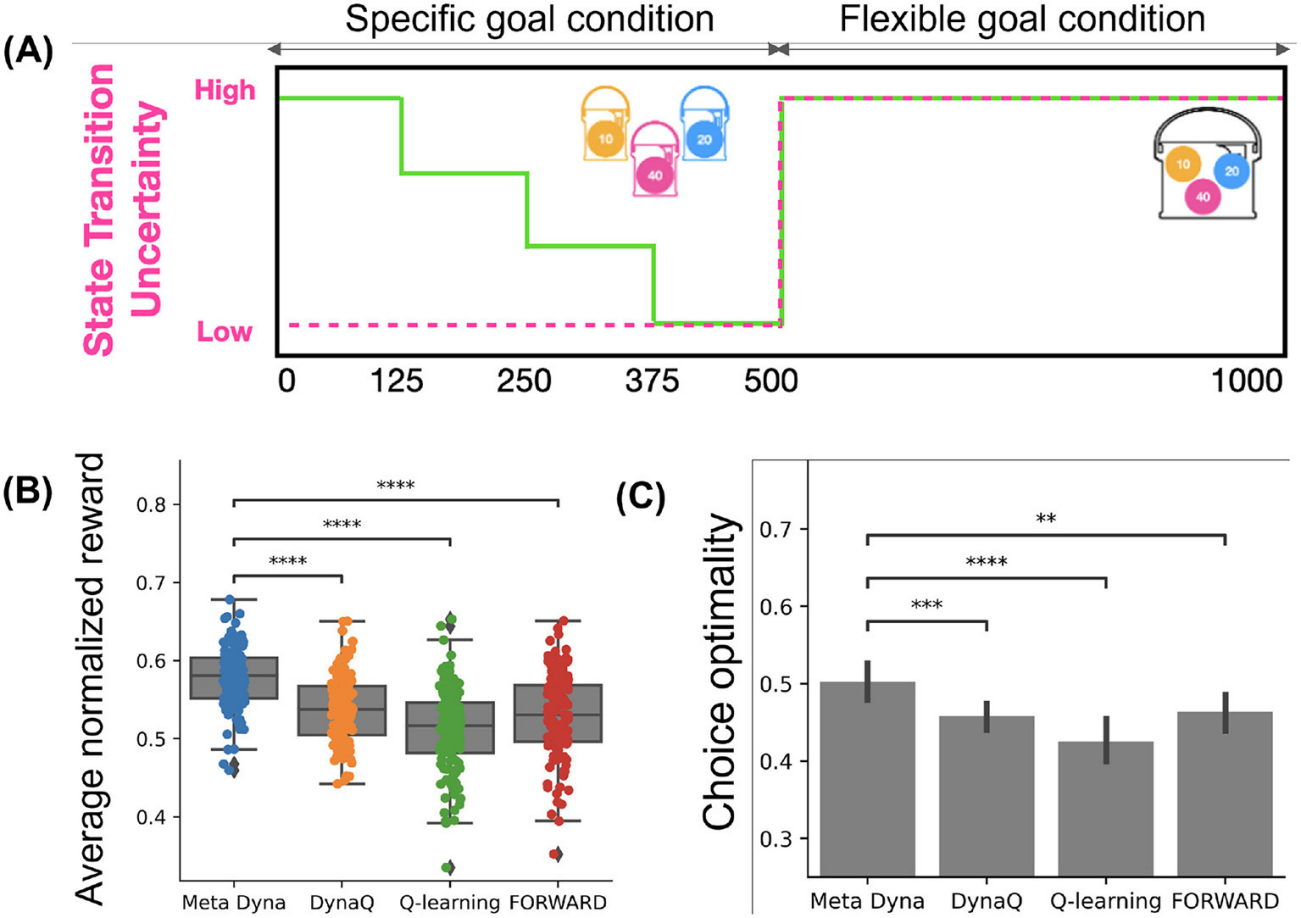
Simulation result. **(A)** Experimental setting. **(B)** Result on average normalized reward. **(C)** Results on choice optimality. (^*^:*p* < 0.05,^**^:*p* < 0.01,^***^:*p* < 0.001,^****^:*p* < 0.0001; independent *t*-test).

The simulation protocol proceeds as follows (Figure 3A). During the initial 500 episodes, the Goal Condition exhibits pseudo-random alterations (uniformly distributed) at fixed 125-episode intervals, maintaining Low State-Transition Uncertainty. This configuration facilitates situations requiring rapid adaptation. The subsequent 500 episodes exhibit high state-transition uncertainty, where goal conditions become less salient, compelling the agent to develop reward-maximizing policies focusing on high-value tokens. This 1,000-episode sequence repeats five times, resulting in 5,000 total episodes, thus enabling comprehensive evaluation of adaptive capabilities across diverse environmental conditions.

#### 4.2.3 Evaluation metrics

The assessment of *Meta-Dyna*'s performance adopted comparative analysis against baseline algorithms: *Dyna-Q, Q-learning*, and *FORWARD*. The reward structure covers a normalized range [0.0, 1.0]. The evaluation metrics includes: (i) mean reward and (ii) choice optimality that quantifies the proportion of episodes where the agent achieves maximum possible reward under specific Goal Conditions (Kim et al., 2019). The choice optimality is formally defined as:


(7) $$\begin{array}{l} \text{Choice Optimality}=\frac{\text{Number of episodes with maximum reward}}{\text{Total number of episodes}}. \end{array}$$

#### 4.2.4 Result

Figure 3 presents the result of the experiment. Figure 3B demonstrates that *Meta-Dyna* achieved superior mean rewards to that of baseline algorithms (*Meta-Dyna*: 0.61, *Dyna-Q*: 0.55, Q-learning: 0.52, FORWARD: 0.54; *p* < 0.0001, independent *t*-test). The clustered distribution of data points suggests *Meta-Dyna*'s robust reward acquisition across environmental variations. The optimality analysis (Figure 3C) shows *Meta-Dyna*'s statistically significant performance on optimal decision over baseline models. This indicates enhanced decision-making capacity under changes in Goal Conditions and State-Transition Uncertainties. These results establish *Meta-Dyna*'s superior performance across all metrics thereby validating the efficacy of meta-control integration within the *Dyna-Q* framework.

We note that the results presented in Figure 3 derive from the tabular implementation of *Meta-Dyna*. Subsequent evaluation using the neural network variant exhibits comparable superiority, with *Meta-Dyna* demonstrating significantly higher mean rewards (*Meta-Dyna*: 0.71, DQN: 0.61, *Dyna-Q*: 0.66; *p* < 0.0001, independent sample *t*-test).

### 4.3 A stochastic *GridWorldLoCA*

#### 4.3.1 Overview

The stochastic *GridWorldLoCA* environment, which originally evaluates MB agents' sample efficiency and adaptive capacity (Wan et al., 2022), exhibits dynamic environmental conditions. This framework comprises distinct phases that are characterized by variations in both initial state distribution (presented as the green region) and reward configurations (presented as black vertical bars at both edges in Figure 2B).

We parameterised the *GridWorldLoCA* environment's complexity through modification of state-transition probabilities, deriving from the Two-Stage MDT. The framework exhibits binary probability distributions—(0.9, 0.1) for the deterministic- and (0.5, 0.5) for the uncertain configuration—which introduce stochastic elements to agent locomotion (Figure 2B). When an agent attempts to move toward a target state, the deterministic configuration assigns 0.9 probability to the intended direction and 0.1 probability distributed across remaining directions. Similarly, in the uncertain configuration, both the intended direction and remaining directions receive equal probabilities of 0.5.

#### 4.3.2 Experimental settings

**Figure 4:**
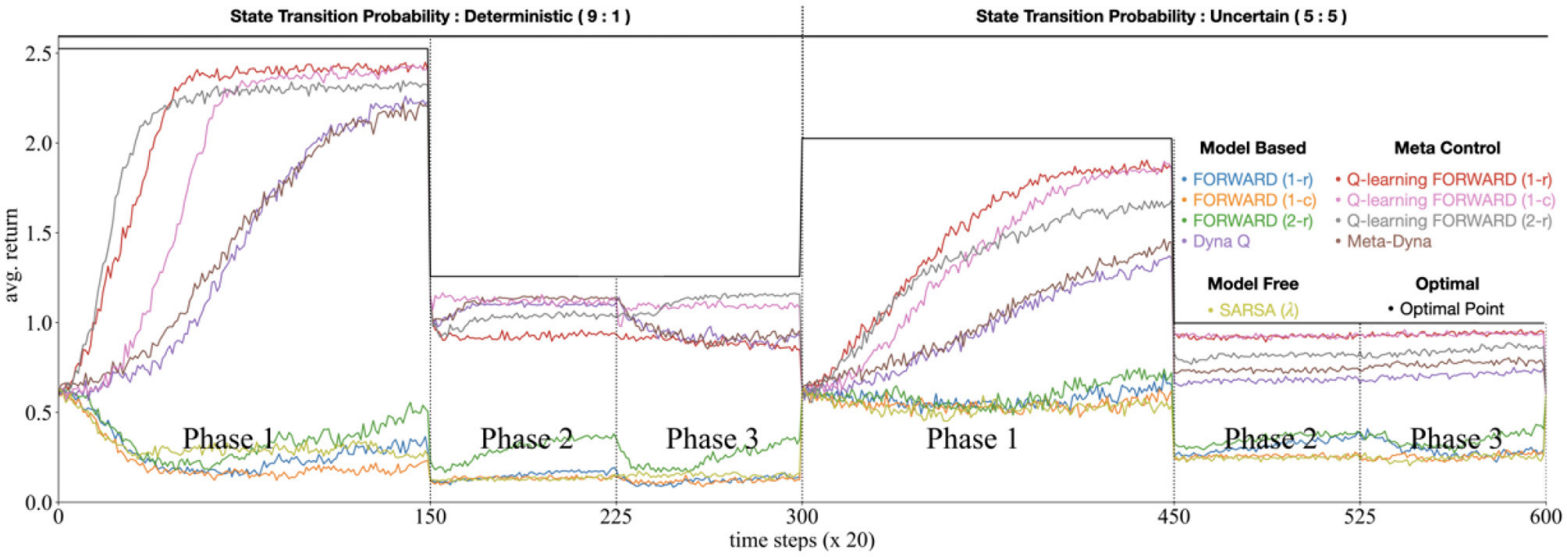
Simulation result on the stochastic *GridWorldLoCA*.

The experimental procedure consists of two blocks each of which has three sequential phases. One block repeats twice during training with distinct state-action-state transition probabilities (please refer to the sequence of phases at the bottom of Figure 4). Each phase introduces novel environmental parameters, characterized by modifications to both the initial state distribution (shown in green) and reward structure (shown in black vertical bars), thus challenging the agent's adaptive capacities.

To rigorously assess adaptive capacity within rapidly changing environments, we implemented a significant reduction in episodic duration per phase—to 3% of the baseline configuration. This constraint intensifies task complexity whilst necessitating accelerated adaptation with limited sampling.

Our experimental evaluation entails comparative analyses across multiple agent architectures: pure MB and MF implementations (FORWARD, *Dyna-Q*, and SARSA, respectively), the proposed *Meta-Dyna*, and hybrid architectures that integrate FORWARD with SARSA or Q-learning. The experimental protocol consists of dual traversals through Phases 1 to 3, with ten complete iterations that establish statistically significant reliability (*p* < 0.05) of performance metrics, including cumulative rewards and policy adaptation speeds.

#### 4.3.3 Result

As illustrated in Figure 4, standalone agents (MB and MF) exhibited suboptimal performance (with the exception of *Dyna-Q*), indicating insufficient adaptability to environmental dynamics. These agents demonstrated particular difficulty in policy adjustment relative to varying initial states and reward structures across experimental phases. Conversely, a family of meta-control agents exhibited rapid adaptation whilst maintaining robust performance throughout all phases.

*Meta-Dyna* demonstrated superior performance, achieving elevated average returns and accelerated optimal policy convergence compared to those of *Dyna-Q* (average return—*Meta-Dyna* > *Dyna-Q*; *p* < 0.05, trial for success—*Meta-Dyna*<*Dyna-Q*; *p* < 0.05, independent *t*-test). These results suggest that *Meta-Dyna*'s meta-control mechanism effectively modulates the integration of MB and MF strategies, thereby facilitating enhanced adaptivity compared to that of *Dyna-Q*. In addition, *Meta-Dyna* exhibits more stable learning behavior (variance: 0.0191) than other models (variance: 0.0733), which allows it to maintain consistency across varying environmental conditions without sacrificing adaptability.

These findings demonstrate that the application of meta-control mechanisms to RL agents brings about significant enhancement in adaptive capabilities compared to standalone implementations. Through effective integration of MB and MF strategies, *Meta-Dyna* achieves rapid policy adaptation in response to environmental dynamics, even under the limited number of training episodes. This experimental evaluation validates *Meta-Dyna*'s efficacy in enhancing sample efficiency and adaptive capabilities within dynamic environments.

### 4.4 Stochastic *Atari Pong*: a probabilistic extension

To evaluate rapid adaptation capacity under the challenges defined in Section 4.1, we developed a *stochastic Atari Pong* environment. This novel framework, which extends the standard Gym, implements state-transition uncertainty and goal-condition dynamics that derive from the Two-Stage MDT.

#### 4.4.1 Experimental setting

Our *stochastic Atari Pong* implementation introduces terminal conditions (episode completion) at single-point acquisition (score of 1), diverging from the traditional 21-point rally structure (Figures 2C, D left). This framework implements two fundamental modifications: state-transition uncertainty and goal condition dynamics.

The state-transition uncertainty manifests through dual mechanisms: paddle locomotion and ball reflection angles. Under deterministic conditions, these parameters maintain high predictability, enabling probabilistic trajectory anticipation (Figure 2D, upper right). Conversely, under stochastic conditions, the introduction of randomness to both parameters necessitates adaptive strategies focused on real-time ball tracking rather than trajectory prediction (Figure 2D, bottom right).

The goal condition framework entails two distinct paradigms. The specific goal condition necessitates ball contact within one of four predetermined paddle segments (uniformly divided quarters of the 20-pixel paddle length, Figure 2D), which results in immediate terminal conditions and reward allocation (+1 for target segment, 0 for others) upon successful execution. In contrast, the flexible goal condition implements standard victory conditions, where reward acquisition occurs upon opponent failure to return the ball.

These environmental parameters are subject to systematic modification (alternating between deterministic and stochastic configurations) at 1,000-timestep intervals throughout the experimental protocol, thus necessitating continuous adaptation to dynamic conditions. This framework requires the RL agent to exhibit rapid adjustment capacity (convergence within 100–200 timesteps) in response to both state-transition uncertainty and goal-condition variations. The experimental evaluation entails a comparative analysis between *Meta-Dyna* and two baseline architectures: DQN and *Dyna-Q*.

In addition, we conducted another experiments to investigate the impact of mental simulation on *Meta-Dyna*'s performance. As described in Section 3.5, *Meta-Dyna* employs a default of 10 mental simulations using the world model, which was maintained across all previous experiments. In this environment, we specifically examined how the number of mental simulations influences performance with respect to average reward (pertaining to the main RL objective) and trials required for success (relating to sample efficiency). The latter metric strongly corresponds to sample efficiency when the RL agent receives a fixed number of real experiences. Specifically, it measures how quickly the agent achieves maximum rewards relative to environmental interactions. We posit that sample efficiency increases as the required number of environmental interactions decreases for achieving maximum reward.

To evaluate this relationship, we parameterised the number of mental simulations and conducted four experimental conditions (*n* = 10, 20, 50, 100) with a fixed number of environmental interactions (i.e., real experiences). We then analyzed the data using the Number of Trials for Success (NTS) metric, defined as:


(8) $$\begin{array}{l} NTS=\frac{R_{max}}{N_{env-interaction}}, \end{array}$$

where *R*_*max*_ denotes the maximum reward achieved by the RL agent, and *N*_*env*−*interaction*_ refers to the number of environmental interactions (equivalent to the number of real experiences).

#### 4.4.2 Results

**Figure 5:**
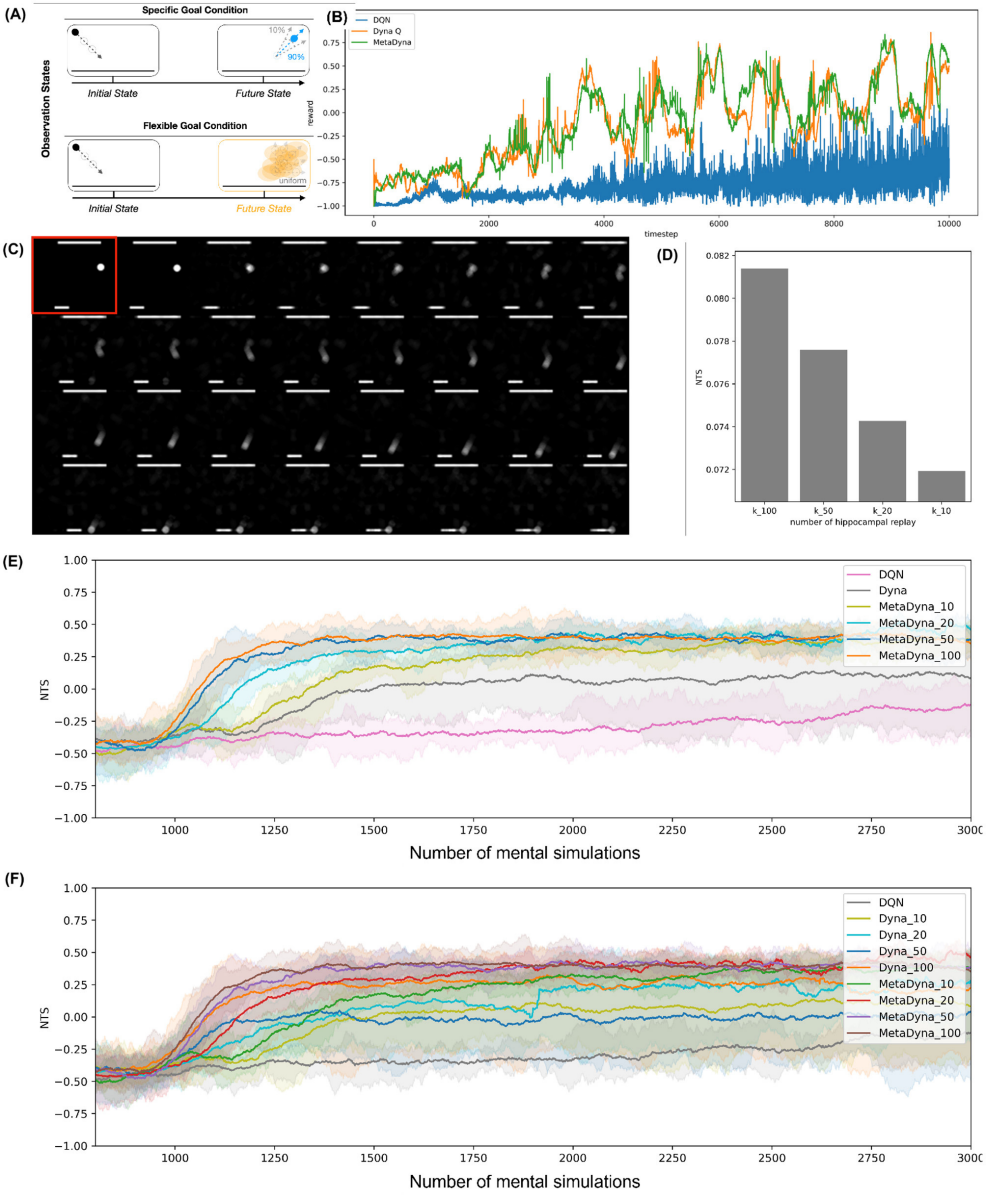
Simulation result on the stochastic *Atari Pong*. **(A)** Two goal conditions coupled with environmental uncertainty. **(B)** Result on three types of RL agents. The X-axis refers to the reward, and the Y-axis refers to the episode. **(C)** The internal representation of the world model. **(D)** Result on the mental simulation. The X-axis refers to the number of mental simulation, and the Y-axis means the number of trials for success (NTS). **(E)** The NTS plot for three types of RL agents. Here, as a baseline model, *Dyna* does not use the function of mental simulation i.e., *n* = 1. *Meta-Dyna* utilizes the function of mental simulation (*n* = 10, 20, 50, 100). **(F)** The NTS plot for three types of RL agents with enabling the function of mental simulation for both *Dyna* and *Meta-Dyna*.

As illustrated in Figure 5, DQN exhibits limited adaptive capabilities (mean reward < 0.4 across all test conditions) within the dynamic environment. The architecture fails to establish policy stability under environmental perturbations (state-transition probability shifts from 0.9 to 0.5 and goal condition alterations), showing only gradual performance improvements after extensive training episodes. This behavior indicates DQN's inherent limitations in rapid adaptation without environmental modeling capabilities.

Both *Meta-Dyna* and *Dyna-Q* exhibit enhanced performance (average policy convergence within 150 timesteps) through their integrated environmental models, which facilitate efficient learning through dynamic environment simulation. Despite similar performance trajectories in the initial training phase (first 1,000 episodes, learning rate ≈ 0.15), *Meta-Dyna* achieves superior cumulative rewards compared with both *Dyna-Q* and DQN (Figure 5B). Quantitative analysis reveals the following cumulative rewards over the entire 5,000-episode evaluation period: Original Reward—*Meta-Dyna*: −0.091, *Dyna-Q*: −0.132, DQN: −0.782; Exponential Reward—*Meta-Dyna*: 0.913, *Dyna-Q*: 0.876, DQN: 0.457 (*p* < 0.001, independent samples *t*-test).

*Meta-Dyna* exhibits superior adaptive capabilities, achieving elevated rewards across all goal conditions. Through effective utilization of its meta-control mechanism, it consistently surpasses both *Dyna-Q* and DQN performance metrics. Despite implementing a more parsimonious parameter structure (single-layer neural network with 128 units compared to LSTM networks with > 500K parameters) compared with sequence-learning architectures such as Meta-RL (Wang et al., 2016), which employ LSTM mechanisms, *Meta-Dyna* achieves comparable or superior cumulative rewards without additional memory architectures. These findings indicate *Meta-Dyna*'s robust generalization capabilities (maintaining > 85% performance across novel environmental configurations with < 200 timesteps adaptation period).

With regards to the impact of mental simulation, Figure 5D illustrates that larger numbers of mental simulations contribute to higher performance given fixed real experiences. This results in two principal conclusions: increasing mental simulations correlates with enhanced performance, and higher numbers of mental simulations improve sample efficiency.

Figure 5E demonstrates *Meta-Dyna*'s superiority in this domain. Whilst baseline models (DQN and *Dyna*) converge to sub-optimal points, all variants of *Meta-Dyna* achieve optimal convergence more rapidly. Moreover, increased mental simulations correlate with faster convergence times and higher reward acquisition. These results indicate that *Meta-Dyna*'s mental simulation mechanism shows promise for improving both environmental adaptivity and sample efficiency.

Figure 5F emphasizes the superiority of *Meta-Dyna*, which implements prefrontal meta-control incorporating mental simulation, compared with the vanilla *Dyna* architecture. As described in Section 2, models of *Dyna* (e.g., *Dyna-Q*) are also equipped with mental simulation capacity. Thus, one might expect performance benefits from increasing *Dyna-Q*'s mental simulations. However, the results reveal a different outcome: even 100 mental simulations of *Dyna-Q* fail to converge toward the optimal point, which *Meta-Dyna* achieves with merely 20 simulations. Furthermore, given equivalent numbers of mental simulations, *Meta-Dyna* consistently outperforms *Dyna-Q*. This discrepancy in performance suggests that prefrontal meta-control contributes significantly to the efficiency differential, despite identical mental simulation functionality. These findings highlight *Meta-Dyna*'s superior sample efficiency and adaptivity through its brain-inspired computational approach.

Overall, the experimental results from our stochastic Atari-Pong environment validate *Meta-Dyna*'s sample efficiency as well as adaptive capacity. Through the incorporation of state-transition uncertainty and goal-condition variability, this framework provided rigorous evaluation of adaptive capabilities within complex, dynamic environments. *Meta-Dyna*'s sustained performance across these conditions substantiates the efficacy of meta-control mechanisms in enhancing RL architectures.

## 5 Discussions and conclusion

This research presents *Meta-Dyna*, a novel prefrontal meta-control framework incorporating mental simulation for RL agents that improves adaptivity and behavioral flexibility. Grounded in neuroscience, it emulates prefrontal cortex-mediated arbitration control mechanisms (Lee et al., 2014) and hippocampal functions (Stachenfeld et al., 2017). The architecture enables both habitual and goal-directed behaviors through integrated Q-learning and planning processes, whilst exhibiting adaptive capacity with sample efficiency through mental simulations under dynamic environmental changes.

The empirical investigation of *Meta-Dyna* entailed three experimental paradigms: the *Two-Stage MDT, Stochastic GridWorldLoCA*, and a *Stochastic Atari-Pong* environment. These frameworks examined cognitive adaptability and computational efficiency through systematic modulation of environmental uncertainty and goal condition dynamics, which derive from the two-stage MDT, an established human decision-making paradigm (Daw et al., 2005; Lee et al., 2014). Within the *Two-Stage MDT, Meta-Dyna* exhibited elevated performance metrics relative to baseline architectures in reward acquisition and choice optimisation.

The *stochastic GridWorldLoCA* evaluation, which examined rapid cognitive adaptation capacity, substantiated the computational efficacy of *Meta-Dyna*. Through its prefrontal meta-control mechanisms, the architecture manifested heightened behavioral flexibility relative to standalone implementations (FORWARD and SARSA). *Meta-Dyna* surpassed *Dyna-Q* in both reward maximization and policy optimisation speed, thus establishing its elevated adaptive capacity.

However, *Meta-Dyna* was not always dominant in the *stochastic GridWorldLoCA*. Compared to a family of *Q-learning FORWARD, Meta-Dyna* demonstrated the lower performance across all phases. In brief, we assume that this phenomenon was caused by differences in implementation methods—direct computation vs. approximation of state-action-state transition probabilities (state-transition probability hereinafter), that is, Tabular RL vs. approximate RL in a broad sense. Although the conceptual framework between these approaches is identical, this implementation difference brings about a discrepancy due to the approximation error which in the end affects the results of the average reward across the stages (for more details, see Supplementary Section 1.1).

The *stochastic Atari-Pong* environment incorporated multidimensional complexity to examine behavioral flexibility and computational efficiency. *Meta-Dyna* manifested proficient arbitration of state-transition uncertainty and variable goal conditions, resulting in elevated performance relative to *Dyna-Q* and DQN architectures. In particular, *Meta-Dyna* exhibited exceptional computational efficiency in experience utilization. The environmental model instantiated dynamic mental simulations, engendering heightened proficiency in both reward accumulation and trial-success speed compared with baseline architectures.

Whilst the environmental model implemented in *Meta-Dyna* enables sequence modeling through MDN-RNN architecture, rapid environmental dynamics lead to sub-optimal sequence predictions. Performance enhancement could be achieved through transformer integration (Vaswani et al., 2017), facilitating more efficient and precise predictions (Radford et al., 2019; Chen et al., 2021). Moreover, the current LSTM-based transition probability approximation within the MDN-RNN framework may benefit from direct probability computation approaches (Hafner et al., 2019). Alternative architectures, such as Recurrent State Space Models (RSSM) (Hafner et al., 2019) or Transformer State Space Models (TSSM) (Chen et al., 2022), could produce enhanced MDP prediction accuracy.

Regarding decision architectures, *Meta-Dyna*'s Q-value foundation could be extended to incorporate recent policy-based achievements, including Trust Region Policy Optimization (TRPO) (Schulman et al., 2015), Proximal Policy Optimization (PPO) (Schulman et al., 2017), and Soft Actor-Critic (SAC) (Haarnoja et al., 2018). Integration of meta-control mechanisms within these frameworks could facilitate rapid optimal action selection under environmental perturbations.

Although *Meta-Dyna* demonstrated superior efficiency metrics relative to baseline models, its single-step environmental model approximation resulted in sub-optimal absolute performance. Implementation of *n*-step approximation, similar to Imagination-Augmented Agents (I2A) (Racanière et al., 2017), could potentially enhance optimality convergence.

The computational cost of mental simulation primarily depends on the number of simulated rollouts. Our analysis shows that increasing the number of mental simulations enhances performance and sample efficiency (Figure 5D). However, increasing the number of rollouts does not guarantee a proportional performance gain relative to the additional computational cost. Considering that the only difference between real experience and mental simulation in our framework is the number of rollouts, we can optimize computational efficiency by dynamically regulating the number of simulations based on task urgency. This adaptive approach ensures that Meta-*Dyna* remains feasible even for time-critical tasks.

Another alternative under time constraint would be the use of an asynchronous approach in which mental simulation is performed by concurrent multiple threads. Like A3C architecture (Mnih et al., 2016), mental simulations in MB RL could be carried out through the parallel execution of hypothetical experiences concurrently, which implements a constant time block to do the rollout. The asynchronous approach offers significant advantages by decorrelating the data into a more stationary process, as parallel agents experience different states simultaneously. This allows for more stable learning while achieving nearly linear speedups with the number of parallel threads employed. Thus, it would be able to achieve the maximum rewards within the time constraint by executing MF with real experiences and MB with simulated experiences. We note that the computational cost in the form of Floating Point Operations Per Second (FLOPS) was not measured in this study, which we will be able to do in the future coupled with an asynchronous approach.

In conclusion, this research presents a neuroscience-inspired RL agent that demonstrates not only rapid environmental adaptation through the parallel implementation of MB and MF strategies, but also exceptional sample efficiency achieved via mental simulation using an RNN-based world model. The agent's architecture, featuring a meta-control mechanism that parallels Acceptance and Commitment Therapy (ACT)'s emphasis on cognitive flexibility (Banerjee et al., 2021), highlights its potential relevance to computational psychiatry and suggests that adaptive regulation may offer valuable insights into cognitive impairments and therapeutic applications (Mei et al., 2025). These findings contribute to our understanding of human RL and the development of biomimetic learning agents. Furthermore, exploring its application in AI-driven cognitive training and behavioral interventions, particularly in the context of digital therapeutics, will be an important avenue for future research (Muyesser et al., 2018; Lee et al., 2024).

Moreover, future investigations will also examine whether Meta-*Dyna*'s mental simulation mechanism can be adapted to model and potentially mitigate maladaptive mental simulation—such as the negative rumination and rigid thought patterns observed in depression (Heo et al., 2021; Senta et al., 2025)—thus providing further insights into pathological cognitive rigidity. Recent work by Heo et al. (2021) demonstrated that depression disrupts arbitration control between MB and MF learning whilst undermining exploitation sensitivity. Similarly, Senta et al. (2025) identified a dual-process impairment in physiological anxiety affecting both reinforcement learning rates and working memory decay. These findings suggest several potential adaptations for Meta-*Dyna*: incorporating variable learning rates that account for altered prediction error processing; modeling impaired arbitration control between simulation strategies; representing working memory constraints; and implementing asymmetric value updating parameters. Such modifications could help simulate how depressive rumination develops when mental simulation becomes trapped in negative feedback loops with diminished capacity to integrate new information or shift between cognitive strategies.

Ultimately, with such computational models of human reinforcement learning, we could provide the means for modeling patients' minds from a computational psychiatry perspective. Once we succeed in this endeavor, we might be able to simulate various treatment regimens using Meta-*Dyna*, which creates opportunities to optimally generate behavioral therapy approaches for patients with precise medication and treatment recommendations.

## Data Availability

The raw data supporting the conclusions of this article will be made available by the authors, without undue reservation.
