# Supplementary material for: Prefrontal meta-control incorporating mental simulation enhances the adaptivity of reinforcement learning agents in dynamic environments
Source: Front Comput Neurosci. 2025 Mar 27;19:1559915. doi: 10.3389/fncom.2025.1559915 (PMC11983510; doi:10.3389/fncom.2025.1559915)
Supplement: Supplementary file 1 [file Data_Sheet_1.pdf]

Supplementary Material

1 SUPPLEMENTARY SECTIONS

1.1 Discrepancy in performance between the family of *Q-learning FORWARD* and *Meta-Dyna*

In brief, we assume that this phenomenon was caused by differences in implementation methods – direct computation versus approximation of state-action-state transition probabilities (state-transition probability hereinafter), that is, Tabular RL versus approximate RL in a broad sense. Although the conceptual framework between these approaches is identical, this implementation difference brings about a discrepancy due to the approximation error which in the end affects the results of the average reward across the stages.

In practice, *Q-learning FORWARD* calculated the state-transition probabilities directly from episodes following the frequentist's approach. As this model functions like an oracle, the *FORWARD* component explicitly computed these probabilities when one episode ended. It therefore maintains a state-transition matrix in the *FORWARD* which explicitly represents the state-transition probability. *Q-learning FORWARD* uses this matrix to infer the next state in response to the action taken during model-based (MB) RL. This results in quick responses to contextual changes in the environment.

In contrast, *Meta-Dyna* relies on a state-value matrix to infer the next state in response to the action, which is embedded in the world model. Unlike *Q-learning FORWARD*, *Meta-Dyna* does not maintain a state-transition matrix that is explicitly computed using episodes. Instead, *Meta-Dyna* constructs a state-value matrix. As this matrix holds the values of each state, the inference of the next state given the current state is straightforward – simply selecting the state that holds a larger value than those in other states. However, each value in the state-value matrix is not equivalent to the state-transition matrix – it is rather an approximated, implicit representation of state-transition than the explicit probability amount, which in turn has potential to possess approximation error. Therefore, there would be likely to perform the erroneous inference about the next state, which hinders precise decision performance over time.

As described in the main manuscript, the mechanism of meta-control is a function of prediction errors (PE). Specifically, the core of meta-control mechanism is the model choice probability, P_{MB}, which is determined by a combination of RPE and SPE. If those two PEs are relatively lower in one implementation than those in the other, the one's P_{MB} will be higher thus the performance would be able to be better than that of the other. Under this assumption, we visualised P_{MB} of two implementations, in *Q-learning FORWARD* and *Meta-Dyna*, across the different phases.

Figure S1 successfully demonstrates what actually happens in P_{MB} between *Q-learning FORWARD* and *Meta-Dyna* across the different phases. The former showed a quicker response to reach to the higher position of P_{MB} against the context changes in the environment than the latter. This is clearly reflected in the performance (Figure S1-A). The explicit computation of state-transition probability in *Q-learning FORWARD* appears superior to the approximated approach in *Meta-Dyna* in terms of adaptivity. In addition, the implementation method affected the level of P_{MB}. The P_{MB} of *Q-learning FORWARD* was consistently higher than that of *Meta-Dyna*. As known, P_{MB} is directly associated with the PEs – the lower the PEs are, the higher the P_{MB} is, thus the higher the overall performance is. As seen in Figure S1-B, *Q-learning*

FORWARD exhibited higher P_{MB} than *Meta-Dyna*, which indicates that the former was not only quicker in adaptation to environmental changes, but also better in learning than the latter.

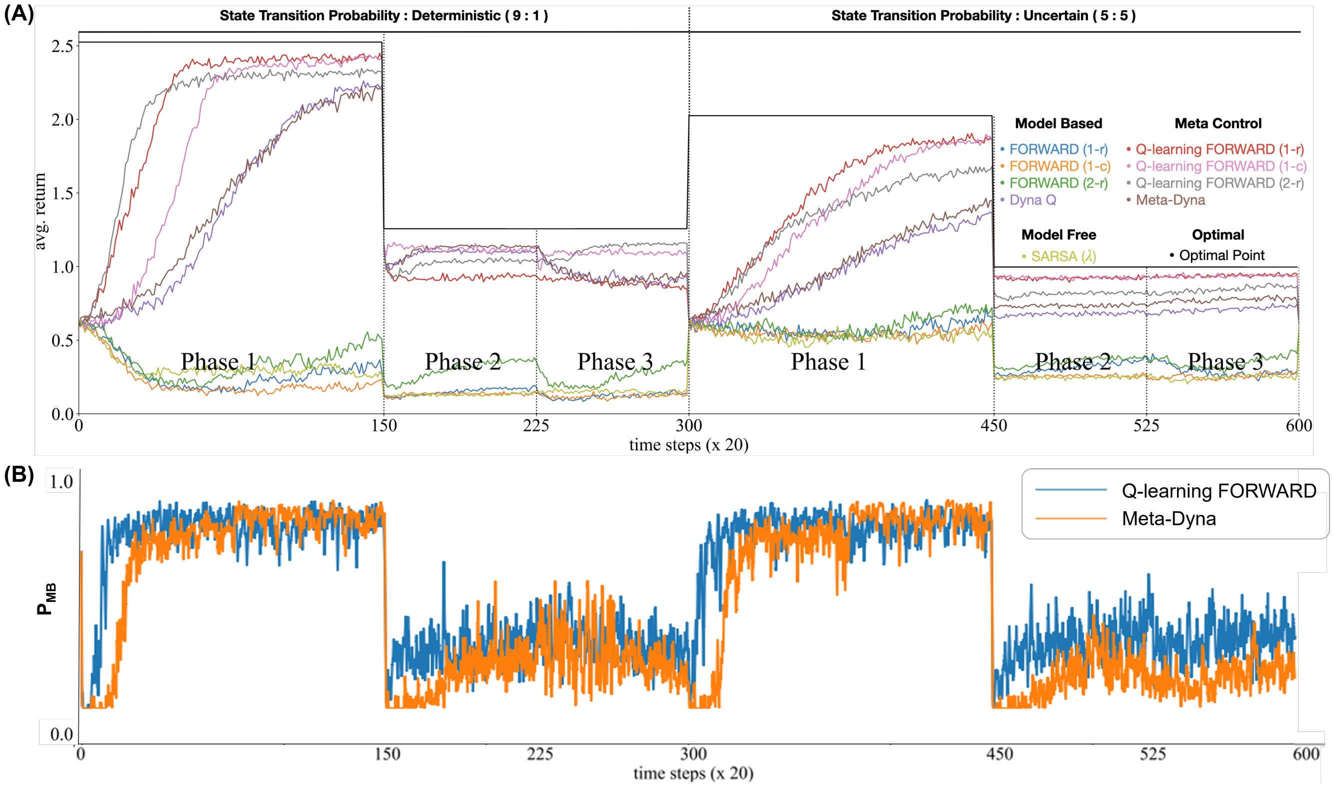

Figure S1. Simulation result on the *stochastic GridWorldLoCA*. (A) Result on the average reward in relation to the context across the different phases. The X-axis represents time steps, and the Y-axis represents the average reward. (B) Changes in P_{MB} across the different phases. The X-axis represents time steps, and the Y-axis represents the average P_{MB}

To examine the relationship between P_{MB} and performance, we presented the Q-values of two implementations shown in Figure S2. It also supports our assumption that the implementation issue affects the outcome of simulations. As seen in Figure S2-A and B, the magnitude of Q-value at each state and the distribution of values are different. The case of *Q-learning FORWARD* is likely to show greater magnitude with a high contrast between states whilst that of *Meta-Dyna* is likely to show lower magnitude with a low contrast, presenting them more blurred. When we compared the two, there were many states whose Q-value was statistically different. Typically, the Q-value of *Q-learning FORWARD* was statistically greater than that of *Meta-Dyna*. As PEs are directly coupled with the performance, this different pattern in Q-value reveals why *Q-learning FORWARD* performs better in the *stochastic GridWorldLoCA* environment than *Meta-Dyna*.

Taken together, we believe that the implementation issue in *Meta-Dyna*, particularly the approximation of state-transition probabilities might affect to the lower performance in the *stochastic GridWorldLoCA* environment. We presume that the same implementation through the frequentist's way to build the state-transition probabilities direct using episodes would bring about the same performance in due course.

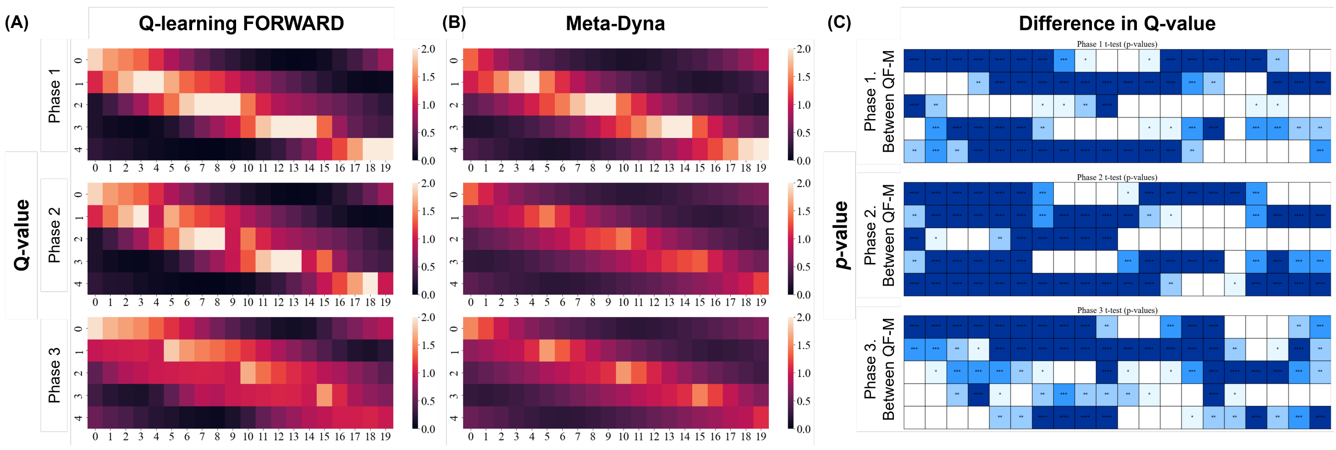

Figure S2. Discrepancy in the pattern of Q-value between *Q-learning FORWARD* and *Meta-Dyna*. (A) Q-value of *Q-learning FORWARD* across the different phases. The X- and Y-axes are X- and Y coordinates of states, respectively. The colour represents the magnitude of Q-value. (B) Q-value of *Meta-Dyna* across the different phases. The X- and Y-axes are X- and Y coordinates of states, respectively. The colour represents the magnitude of Q-value. (C) Statistical differences between Q-value of *Q-learning FORWARD* and that of *Meta-Dyna*. The X- and Y-axes are X- and Y coordinates of states, respectively. The colour represents the degree of statistical significance.

2 SUPPLEMENTARY FIGURES

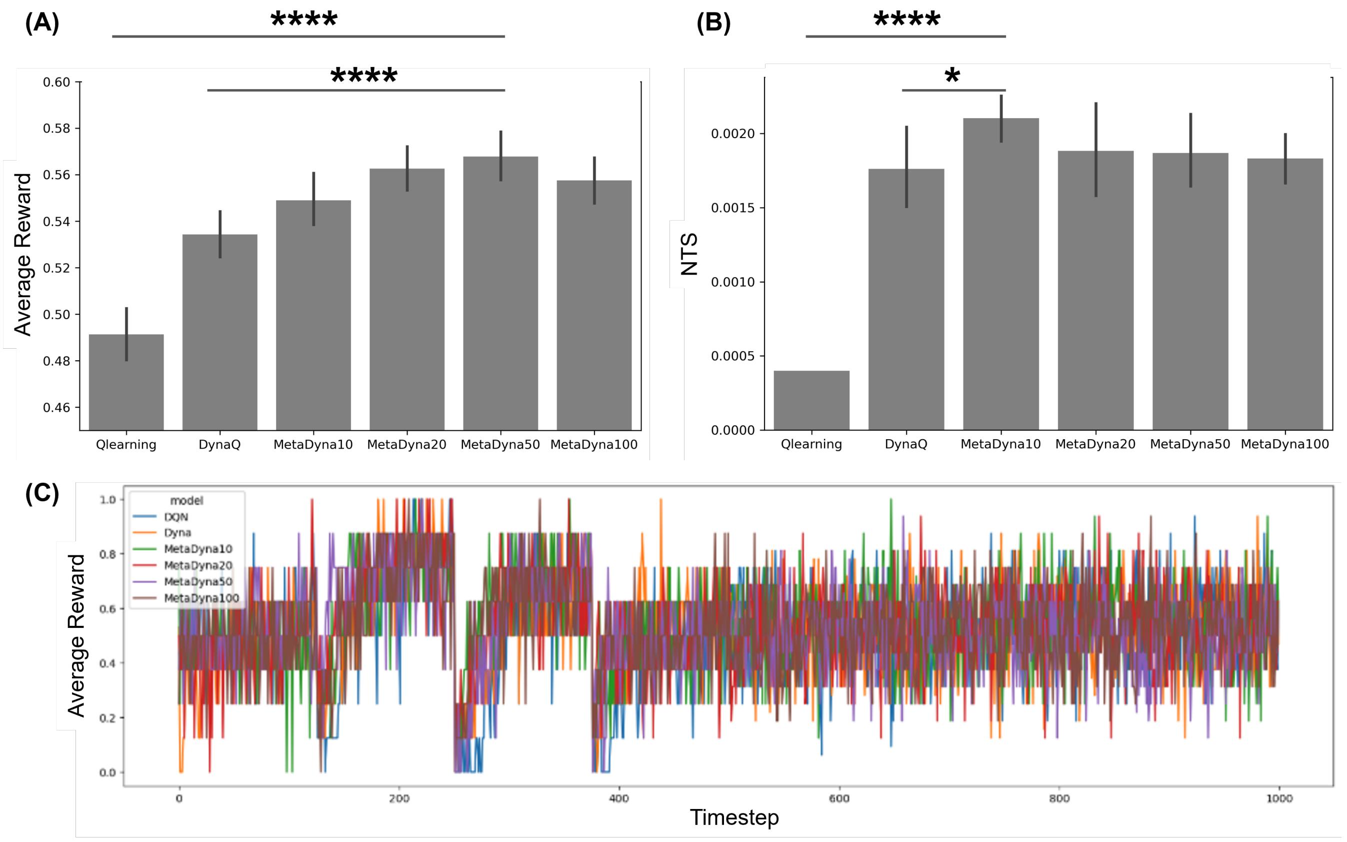

Figure S3. Simulation result on two-stage Markov decision task. (A) Result on the average reward across Meta-Dyna, Dyna and Q-learning. (B) Result on the NTS across Meta-Dyna, Dyna and Q-learning. $* : p < 0.05, ** : p < 1e-2, *** : p < 1e-3, **** : p < 1e-4$ (independent t-test).
